# Reconstructed Human Skin Models to Study Superficial and Deep Skin Wound Healing In Vitro

**DOI:** 10.1111/wrr.70047

**Published:** 2025-06-05

**Authors:** Maaike Waasdorp, Irit Vahav, Joline Paulina Nugteren‐Boogaard, Sanne Roffel, Susan Gibbs

**Affiliations:** ^1^ Department of Molecular Cell Biology and Immunology, Amsterdam Institute for Infection and Immunity Amsterdam UMCs Location VUmc Amsterdam the Netherlands; ^2^ Center for Translational Immunology University Medical Center Utrecht, Utrecht University Utrecht the Netherlands; ^3^ Department of Oral Cell Biology, Academic Center for Dentistry Amsterdam (ACTA) University of Amsterdam and Vrije Universiteit Amsterdam Amsterdam the Netherlands

**Keywords:** adipocytes, in vitro, organotypic, re‐epithelialisation, skin, stromal cells, tissue engineering, wound healing

## Abstract

Wound healing is an essential and complex biological mechanism to repair barrier breaches in the human body, but it results in scar formation. The extent of scar formation is associated with the depth of injury. Stromal cells play a vital role in wound healing and scar formation, but the role of the subcutaneous tissue in human skin wound healing remains largely unknown. In order to dissect the role of dermal fibroblasts, adipose stromal cells, and adipocytes in superficial and deep skin wound healing, we created a human tissue‐engineered skin model and assessed healing outcomes in vitro. Three different reconstructed skin models were created, with dermal fibroblasts, adipose stromal cells, or adipocytes in the wound bed underneath a standardised biopsy punch wound. The superficial skin wound model with only dermal fibroblasts in the wound bed was completely healed within 14 days. The engineered ‘deep’ wounds with adipocytes in the wound bed showed delayed wound closure with reduced Ki67 proliferating keratinocytes and reduced basement membrane collagen IV deposition. This was accompanied by increased wound contraction and α‐SMA protein expression underneath the newly formed epidermis, indicative of early scar formation. The ‘deep’ wound model with adipose stromal cells but without adipocytes showed improved re‐epithelialisation but still healed with increased α‐SMA protein expression. Furthermore, decreased leptin was observed in the supernatant of the ‘deep’ wound model. The superficial and deep wound models presented here can be used to test future therapies to improve wound closure which will lead to improved scar formation.

**Abbreviations**
Adi: adipocytes
ADM: adipocyte differentiation medium
ALI: air‐liquid‐interface
AMM: adipocyte maintenance medium
ASC: adipose stromal cells
DAMPs: damage associated molecular patterns
Fib: fibroblasts
KC: keratinocyte
RhS: reconstructed human skin

## 1 Introduction

Skin wound healing is an essential and complex biological mechanism to repair barrier breaches in the human body. Timely repair is of vital importance to prevent infections, but usually results in the formation of scar tissue with reduced functional and barrier properties. Scarring is associated with injury depth [1]. Superficial wounds, such as a graze or split skin graft donor site, usually heal well, with normotrophic (thin and flat) scars that gradually fade over time. However, deep wounds occurring after extensive trauma or burn injury often heal with excessive or adverse scar formation, also known as hypertrophic scars [2]. Hypertrophic scars are contractile, red, raised, itchy scars that can reduce joint mobility and therefore severely impact quality of life [3]. Therefore, adverse scar formation is associated with a physical as well as a psychological burden to patients and society. To date, hypertrophic scars are mainly treated with corticosteroid injections, 5‐fluorouracil (5‐FU) injections, cryotherapy, or laser therapy, and more efficient treatment options are eagerly awaited. A major limitation in finding such treatment options is the lack of physiologically relevant human models.

Current models that are used to study deep wound healing and scar formation include animal models, excised animal or human skin, or in vitro cultured cells or skin equivalents (also known as reconstructed human skin or RhS) [4, 5]. Mouse models are most commonly used to study wound healing, but mouse skin poorly resembles human skin physiology. For example, wounds in mice heal primarily by contraction, mice do not develop hypertrophic scars, and the anatomical architecture of skin and underlying fat and muscle layers is very different [6]. Primary human skin cells are relatively easy to harvest and cultivate, and standardised assays such as proliferation or wound scratch assays allow for high‐throughput screening of potential drug candidates. These models, however, do not take into account the complexity of skin wound healing that is needed to more accurately predict clinical outcome. In vitro RhS models allow for selective and reproducible incorporation of different cell types into a construct that is entirely of human origin, thus enabling simultaneous read‐out of multiple parameters (e.g., combination of dermal and epidermal parameters and cytokine secretion) associated with re‐epithelialisation and early wound healing [7, 8].

The difference in wound healing and scar formation between deep and superficial wounds may be attributed to the different types of (stromal) cells inside the wound bed. Stromal cells and myofibroblasts specifically play a vital role in wound healing by replacing and reconstructing the damaged stromal tissue via the production of extracellular matrix and wound site contraction. Excessive contraction and deposition of extracellular matrix, however, result in the formation of a fibrous, hypertrophic scar tissue. Recent studies revealed heterogeneity in stromal cell populations in skin and their specific role in wound healing and scar formation [9]. Whereas papillary fibroblasts that reside in the upper part of the dermis are associated with re‐epithelialisation and hair follicle formation, fibroblasts from the deeper reticular dermis facilitate the initial formation of granulation tissue and production of extracellular matrix [10]. Stromal cells from the subcutaneous fat layer, also known as adipose stromal or stem cells (ASCs) or fascia fibroblasts, stimulate wound healing but also increase contraction and scar formation [8, 11, 12]. Moreover, adipocytes are a potential source for novel fibroblasts and myofibroblasts and have been shown to stimulate the healing of deep wounds in mice [13]. Recently, we have described the incorporation of an adipose layer beneath our RhS model consisting of differentiated epidermis on a fibroblast‐populated collagen hydrogel and found that the adipose layer contributed to increased metabolic activity and reduced inflammatory state that is beneficial for tissue homeostasis and possibly also tissue repair [14]. In general, fibroblasts from deeper layers of the skin contribute to scar formation, but the role of adipocytes in wound healing is not yet elucidated. It is currently unknown how ASC, combined with adipocytes, contribute to adverse scar formation.

Current in vitro tissue‐engineered wound healing models do not take into account the deeper (subcutaneous) layer, and existing models that include a subcutaneous fat layer do not incorporate standardised open wounds, distinguishing superficial dermal wounds from deep wounds extending into the adipose layer [15, 16, 17, 18, 19, 20]. In order to investigate the role of the subcutaneous tissue in wound closure and scar formation, we created RhS models that include the epidermis and dermal layer as well as a subcutaneous adipose layer containing adipocytes and/or ASCs. To mimic a superficial wound that heals over an intact dermis, the in vitro wound bed consisted only of dermal fibroblasts (RhS‐fib). Deep wounds where the dermis is completely removed in the wounded area were mimicked in RhS‐ASC and RhS‐adi, where the wound bed consists of respectively ASCs and ASCs+adipocytes. By comparing wound healing over time in RhS‐fib, and in particular between RhS‐ASC and RhS‐adi, more insight into the role of ASCs and adipocytes in deep wound healing outcome was obtained.

## 2 Materials and Methods

### 2.1 Cell Isolation and Culture

Normal skin (including subcutaneous adipose tissue) was obtained from patients undergoing abdominal dermolipectomy or breast reduction surgery and collected anonymously. Skin tissue was collected after obtaining informed consent, and collection procedures were in compliance with the ‘Code for Proper Secondary Use of Human tissue’ as formulated by the Dutch Federation of Medical Scientific Organisation.

Keratinocytes, fibroblasts, and ASCs were isolated from skin and subcutaneous adipose tissue, respectively, as described previously [21, 22]. In short: adipose tissue was removed from the dermis and epidermis, cut into pieces, and washed thoroughly with PBS to remove most erythrocytes. Adipose tissue was incubated in collagenase type II (7.5 mg/mL; Gibco, Invitrogen, Paisly, UK)/dispase II (0.6 mg/mL; Roche, Mannheim, Germany) solution for 2 h at 37°C. The remaining epidermis and dermis were cut into 1 × 2 mm pieces and incubated in 2.4 mg/mL dispase II at 4°C. After overnight incubation, dermis and epidermis were separated and epidermal sheets were incubated in 0.05% trypsin/0.001% EDTA for 15 min. Dermis was incubated in collagenase type II/dispase II solution for 2 h at 37°C. All cells were passed through 100 and 40 μm cell strainers. Fibroblasts and ASCs were cultured in a 37°C, 5% CO_2_ atmosphere in DMEM (BE12‐604F, Lonza) supplemented with 1% penicillin/streptomycin (Invitrogen, Carlsbad, CA, USA) and 1% UltroserG (BioSepra SA, Cergy‐Saint‐Christophe, France). Keratinocytes were cultured on collagen IV (0.5 mg/cm^2^; Sigma‐Aldrich) coated culture plates in DMEM/HAM's F12 (3:1) supplemented with 1% penicillin/streptomycin (Invitrogen, Carlsbad, CA, USA), 1% UltroserG, 0.86 nM insulin (Sigma‐Aldrich), 1 μM hydrocortisone (Sigma‐Aldrich), 1 μM isoproterenol (Sigma‐Aldrich), and 2 ng/mL KGF (Sigma‐Aldrich) in an incubator at 37°C, 7.5% CO_2_ atmosphere.

ASCs were differentiated into adipocytes at passage 3–4 [21, 23] and differentiation was confirmed by light microscopy (fat droplet accumulation) and leptin and adiponectin secretion (Figure S1). Two days after reaching confluence, adipogenic differentiation was induced using differentiation medium (DMEM/HAM's F12 1:1 supplemented with 1% penicillin/streptomycin, 33 μM d‐Biotin (Sigma‐Aldrich), 17 μM D‐Pantothenic acid humicalcium sal (Sigma‐Aldrich), 100 nM dexamethasone (Sigma‐Aldrich), 100 nM insulin (Humulin R; Lilly, VUmc Pharmacy), 1 μM Rosiglitasone (Sigma‐Aldrich), 500 μM IBMX (Sigma‐Aldrich), 2 nM T3 and 10 μg/mL Transferrin (Sigma‐Aldrich)). After 3 days of differentiation, the medium was changed to maintenance medium (DMEM/Ham's F12 1:1 supplemented with 1% penicillin/streptomycin, 33 μM d‐Biotin, 17 μM D‐pantothenic acid hemicalcium sal, 10 nM dexamethasone and 10 nM insulin (humulin R)). Five days after the initiation of differentiation, when 60%–80% of cells contained visible fat droplets, (pre)adipocytes were used for the construction of the RhS‐adi 3D wound models.

### 2.2 Construction of Reconstructed Human Skin (RhS) Wound Models

**FIGURE 1:**
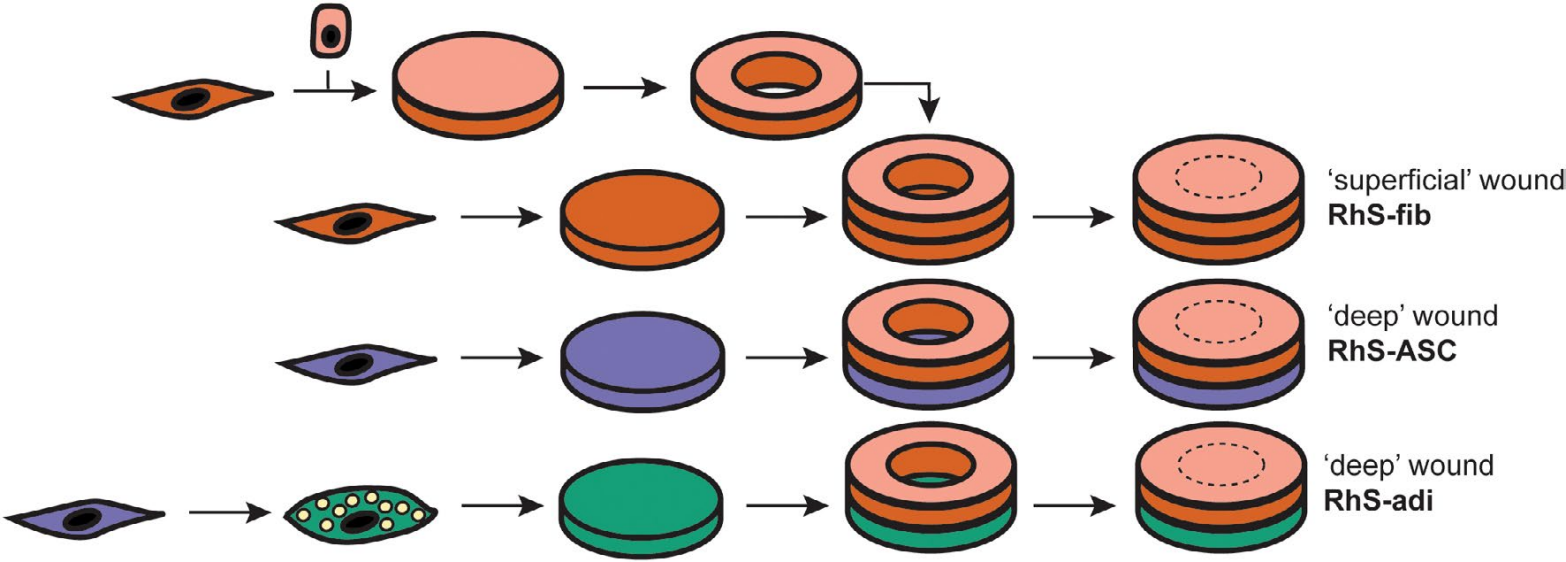
Construction of superficial and deep wound models RhS‐fib, RhS‐ASC and RhS‐adi. Schematic representation of RhS‐fib (a), RhS‐ASC (b) and RhS‐adi (c) wound construction and the culture media used in each step of the construction process. Adi, adipocyte; ADM, adipocyte differentiation medium; ALI, air‐liquid interface; ASC, adipose stromal cell; fib, fibroblast; KC, keratinocyte; RhS, reconstructed human skin.

RhS were constructed from donor‐matched keratinocytes (passage 1–2), fibroblasts (passage 2–3), adipose stromal cells (passage 2–3) and/or (pre‐)adipocytes (passage 3–4). A schematic presentation of RhS‐fib, RhS‐ASC and RhS‐adi wound construction is shown in Figure 1 and an overview of culture media used throughout the process is provided in Table 1. First, dermal layers were prepared by seeding 4 × 10^5^ fibroblasts onto 2.2 × 2.2 cm squares of matriderm (Dr. Suwelack Skin & Health Care, Germany) and cultured submerged for 3 weeks at 37°C, 5% CO_2_ in DMEM/Ham's F12 (3:1) supplemented with 1% penicillin/streptomycin, 2% Ultroser G, 86 nM insulin, 65 μg/mL ascorbic acid and 5 ng/mL EGF (Sigma‐Aldrich). Next, wound bed layers were prepared by seeding 4 × 10^5^ fibroblasts, 4 × 10^5^ ASCs, or 3 × 10^6^ pre‐adipocytes onto 2.2 × 2.2 cm squares of matriderm. Fibroblast and ASC cultures were kept in DMEM/Ham's F12 (3:1) supplemented with 1% penicillin/streptomycin, 2% Ultroser G, 86 nM insulin, 65 μg/mL ascorbic acid and 5 ng/mL EGF (Sigma‐Aldrich) for 3 weeks. Adipocyte cultures were grown in adipocyte maintenance medium for 3 weeks to stimulate further maturation of adipocytes. 5 × 10^5^ keratinocytes were seeded on top of each dermal layer (3‐week‐old fibroblast‐populated matriderm) and cultured for 3–4 days submerged in 0.4 μm pore size transwell inserts (Costar Corning Inc., New York, NY, USA) in normal keratinocyte medium at 37°C, 7.5% CO_2_. Cultures were lifted to the air‐liquid interface (ALI) using 6 well deep well culture plates (Corning), and cultured in ALI culture medium (DMEM/Ham's F12 (3:1) supplemented with 1% penicillin/streptomycin, 0.2% Ultroser G, 100 nM insulin, 1 μM hydrocortisone, 1 μM isoproterenol, 10 mM L‐serine (Sigma‐Aldrich), 10 μM L‐carnitine (Sigma‐Aldrich), 25 μM palmitic acid (Sigma‐Aldrich), 15 μM linoleic acid (Sigma‐Aldrich), 7 μM arachidonic acid (Sigma‐Aldrich), 1 μM vitamin E (Sigma‐Aldrich) and 65 μg/mL ascorbic acid (Sigma‐Aldrich)) for an additional 7 days allowing epidermal differentiation. After 7 days of air‐exposed culture, a full thickness wound was made into the RhS by piercing the epidermis and entire dermal layer using a 4 mm diameter biopsy punch (Kai Medical) and each wounded construct was placed on top of a wound bed layer containing either fibroblast (RhS‐fib), ASC (RhS‐ASC), or adipocytes (RhS‐adi), that at this point have been pre‐cultured in their respective medium for 3 weeks. After assembly of the wounds, all constructs were cultured in ALI culture medium and allowed to heal for up to 14 days. Culture medium was refreshed and collected for protein analysis at 1, 4, 7, 10 and 14 days after construction of the wounds. At 1, 4, 7 and 14 days after wounding, 2 cultures of each type (RhS‐fib, RhS‐ASC and RhS‐adi) were processed for histology and immunohistochemical evaluation. The RhS tissue was cut in half, precisely through the middle of the wound. If it was macroscopically suspected that the wound was unevenly covered, the cut was made in such a way that both sides would be represented in the cross‐section.

**TABLE 1:** Overview of culture media.

	Basal medium	Additives
Fibroblast 2D medium (F2D)	DMEM	1% penicillin/streptomycin and 1% Ultroser G
Fibroblast 3D medium (F3D)	DMEM:Ham's F12 (3:1)	1% penicillin/streptomycin, 2% Ultroser G, 86 nM insulin, 65 μg/mL ascorbic acid and 5 ng/mL EGF
Keratinocyte 2D medium (KC1)	DMEM:Ham's F12 (3:1)	1% penicillin/streptomycin, 1% Ultroser G, 86 nM insulin, 1 μM hydrocortisone, 1 μM isoproterenol, and 2 ng/mL KGF
Adipocyte differentiation medium (ADM)	DMEM:Ham's F12 (1:1)	1% penicillin/streptomycin, 33 μM d‐Biotin, 17 μM D‐Pantothenic acid humicalcium sal, 100 nM dexamethasone, 100 nM insulin, 1 μM Rosiglitasone, 0.5 mM IBMX, 2 nM T3 and 10 ug/mL Transferrin
Adipocyte maintenance medium (AMM)	DMEM:Ham's F12 (1:1)	1% penicillin/streptomycin, 33 μM d‐Biotin, 17 μM D‐pantothenic acid hemicalcium sal, 10 nM dexamethasone and 10 nM insulin
Air‐liquid‐interace medium (ALI)	DMEM:Ham's F12 (3:1)	1% penicillin/streptomycin, 0.2% Ultroser G, 100 nM insulin, 1 μM hydrocortisone, 1 μM isoproterenol, 10 mM L‐serine, 10 μM L‐carnitine, 25 μM palmitic acid, 15 μM linoleic acid, 7 μM arachidonic acid 1 μM vitamin E and 65 μg/mL ascorbic acid

### 2.3 Wound Contraction

Photographs were taken at each medium refreshment to determine wound contraction. Wound contraction was determined by measuring the initial surface of the wound at *t* = 0 and *t* = 14 using ImageJ software. Contraction was calculated as follows: [initial wound surface area at day 14]/[initial wound surface at day 0] × 100%.

### 2.4 Histology and Immunohistochemistry

Formalin‐fixed paraffin embedded tissue sections (5 μm) were used for histological analysis (haematoxylin & eosin staining) and immunohistochemical staining with the following antibodies: Mouse‐anti‐collagen IV (1:100, clone 1042, Mon3251, Monosan), mouse‐anti‐ki67 (1:50, clone Mib1, M7240, Dako), Mouse‐anti‐α‐SMA (1:200, clone 1a4, M0851, Dako), mouse‐anti‐vimentin (1:200, clone V9, M0725, Dako), mouse‐anti‐K10 (1:1000, clone DE‐K10, 11414, Progen), mouse‐anti‐K15 (1:50, clone LHK15, Monx10690, Monosan), mouse‐anti‐perilipin (1:1, clone PERI 112.17, 651156, Progen), rabbit‐anti‐ITGA5 (1:500, HPA002642, Sigma‐Aldrich). In short, 5 μm tissue sections were deparaffinised and boiled in citrate buffer pH 6.0 (ki67, collagen IV, vimentin, ITGA5) or Tris‐EDTA buffer pH 9.0 (perilipin) for 20 min or incubated with pepsin (GM302, Dako, Agilent Technologies) for 5 min at 37°C (collagen IV, keratin 10, keratin 15), prior to incubation with the primary antibody for 1 h at room temperature. Slides were incubated with BrightVision 2‐step detection system anti‐mouse/Rabbit HRP (VWRKDPVB110HRP, Immunologic), followed by 3‐amino‐9‐ethylcarbazole (AEC, Sigma‐Aldrich) substrate staining and counterstaining using haematoxylin. Pictures were taken at 20 times magnification using a Nikon Eclipse 80i microscope (Düsseldorf, Germany) or a Vectra Polaris slide scanner (PerkinElmer Inc.).

### 2.5 ELISA

For protein quantification in culture supernatants, ELISA reagents were used in accordance with the manufacturer's specifications. For CXCL8/IL‐8, a human IL‐8 ELISA kit was used (Sanquin, Amsterdam, the Netherlands). For leptin (DY398), CCL27 (BAF376) and CXCL10 (BAF266), a DuoSet was used (R&D System Inc. Minneapolis, Minnesota). Paired ELISA antibodies and recombinant proteins were used for IL‐6 (BAF206; MAB206; 206‐IL‐010), CCL2 (MAB679; BAF279), CCL5 (278‐RN‐010; BAF278), VEGF (BAF293; AF‐293‐NA; 293‐VE‐10) and TIMP‐1 (MAB970; 970‐TM; BAF970) (all from R&D System Inc., Minneapolis, Minnesota).

### 2.6 Data Analysis and Statistical Analysis

Six independent experiments were performed, each from cells isolated from a different donor. For each independent experiment, at least one intra‐experiment duplicate (depending on primary cell availability) was included to take into account technical variation. In total, 15 RhS‐fib, 15 RhS‐ASC, and 9 RhS‐adi wounded cultures were analysed from 6, 6, and 4 different donors, respectively. Data on RhS‐adi models from 2 donors are missing due to limited adipocyte availability. Measures from technical replicates were averaged prior to analysis. All values are expressed as mean ± SD. All groups were tested for normality using the D'Agostino‐Pearson omnibus normality test. For multiple comparisons, one‐way‐ANOVA analysis (for parametric) or Kruskal‐Wallis or Friedman test (for matched or unmatched nonparametric values) were used, followed by Bonferroni's or Dunn's multiple comparison tests, respectively. In case of missing values, the (matched) data were analysed by fitting a mixed model with Geisser–Greenhouse correction, followed by Tukey's multiple comparison test. All analyses were performed using GraphPad Prism version 9.1.0.

Re‐epithelialisation was determined by measuring the total wound diameter and total length of the re‐epithelialised area in HE‐stained tissue sections using NIS elements AR 3.2 software (Nikon, Tokyo, Japan). Ki67 positive and negative cells in the basal layer were counted, and proliferation was expressed as % ki67 positive cells in immuno‐stained tissue sections. The collagen IV and α‐SMA positive area was determined over the entire length of re‐epithelialised wound area in immuno‐stained tissue sections using Qupath software v0.2.0‐m7 [22].

## 3 Results

### 3.1 Reconstruction of Full Thickness RhS Containing Keratinocytes, Fibroblasts, Adipose Stromal Cells and Adipocytes

**FIGURE 2:**
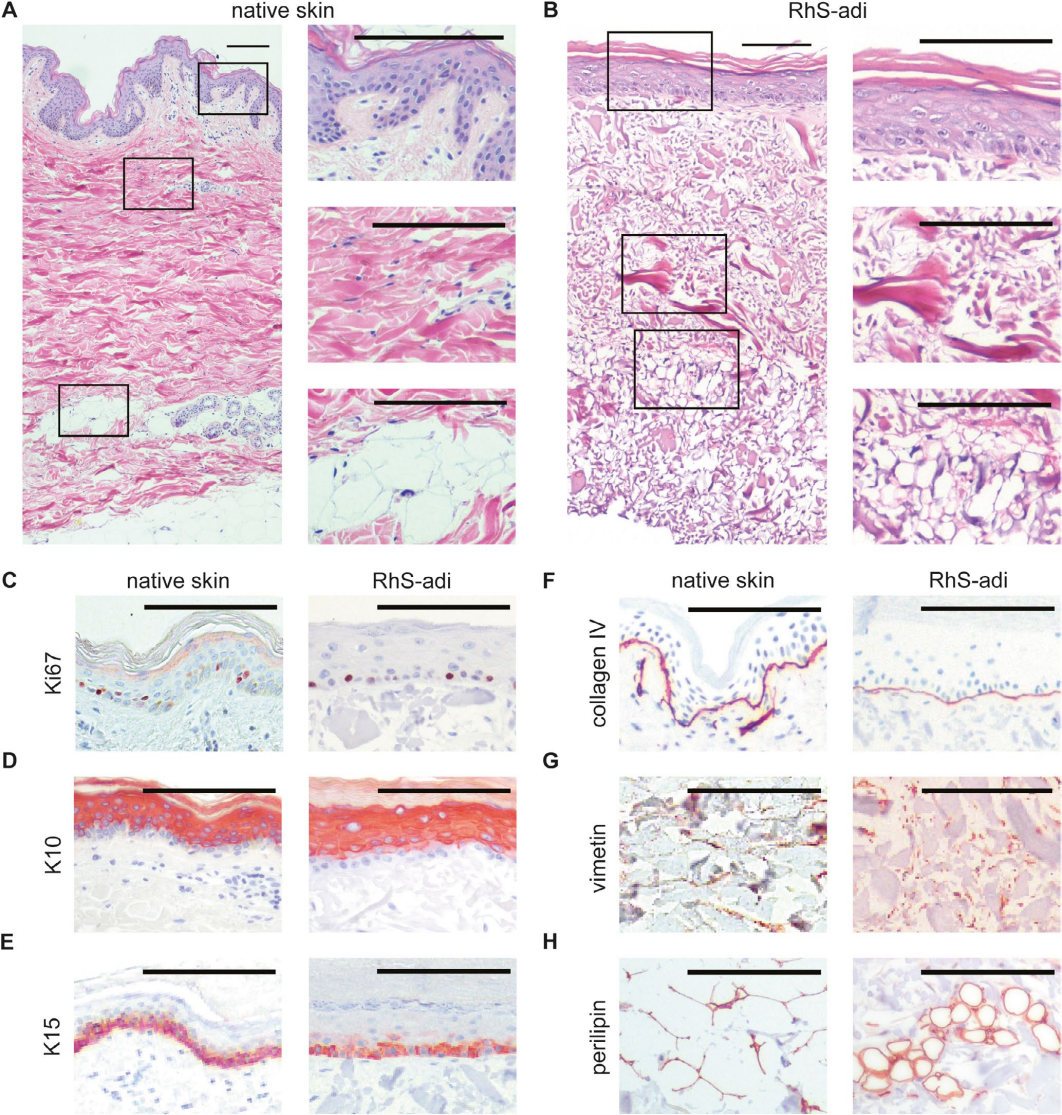
Histological characteristics of the three‐layered reconstructed human skin. Haematoxylin and eosin staining of (A) native human skin and (B) RhS‐adi with magnification of epidermis, dermis and subcutis. Immunohistochemical staining of characteristics of native human skin and RhS‐adi showing the markers (C) ki67 for proliferation, (D) K10 for differentiated keratinocytes, (E) K15 for basal layer keratinocytes, (F) collagen IV for basement membrane, (G) vimentin for stromal cells and (H) perilipin for adipocytes. All scale bars represent 100 μm.

After 14 days of culture at the ALI, the 3D full thickness RhS‐adi consisted of a stratified epithelium on top of a fibroblast‐populated dermis and an ASC and adipocyte‐populated hypodermis layer, representing normal human skin (Figure 2). Similar to native skin, the stratified epidermis contained a K15‐positive basal layer with ~18% Ki67^+^ proliferating keratinocytes and multiple K10‐positive suprabasal layers. The epidermis was separated from the dermal compartment by a collagen IV‐positive basement membrane. The subcutaneous layer contained both vimentin‐positive ASCs and perilipin‐positive ASC‐derived adipocytes, which appeared to have a white‐adipocyte morphology, containing one big lipid droplet per cell.

### 3.2 Re‐Epithelialisation in ‘Superficial’ and ‘Deep’ Wounds With and Without Adipocytes

**FIGURE 3:**
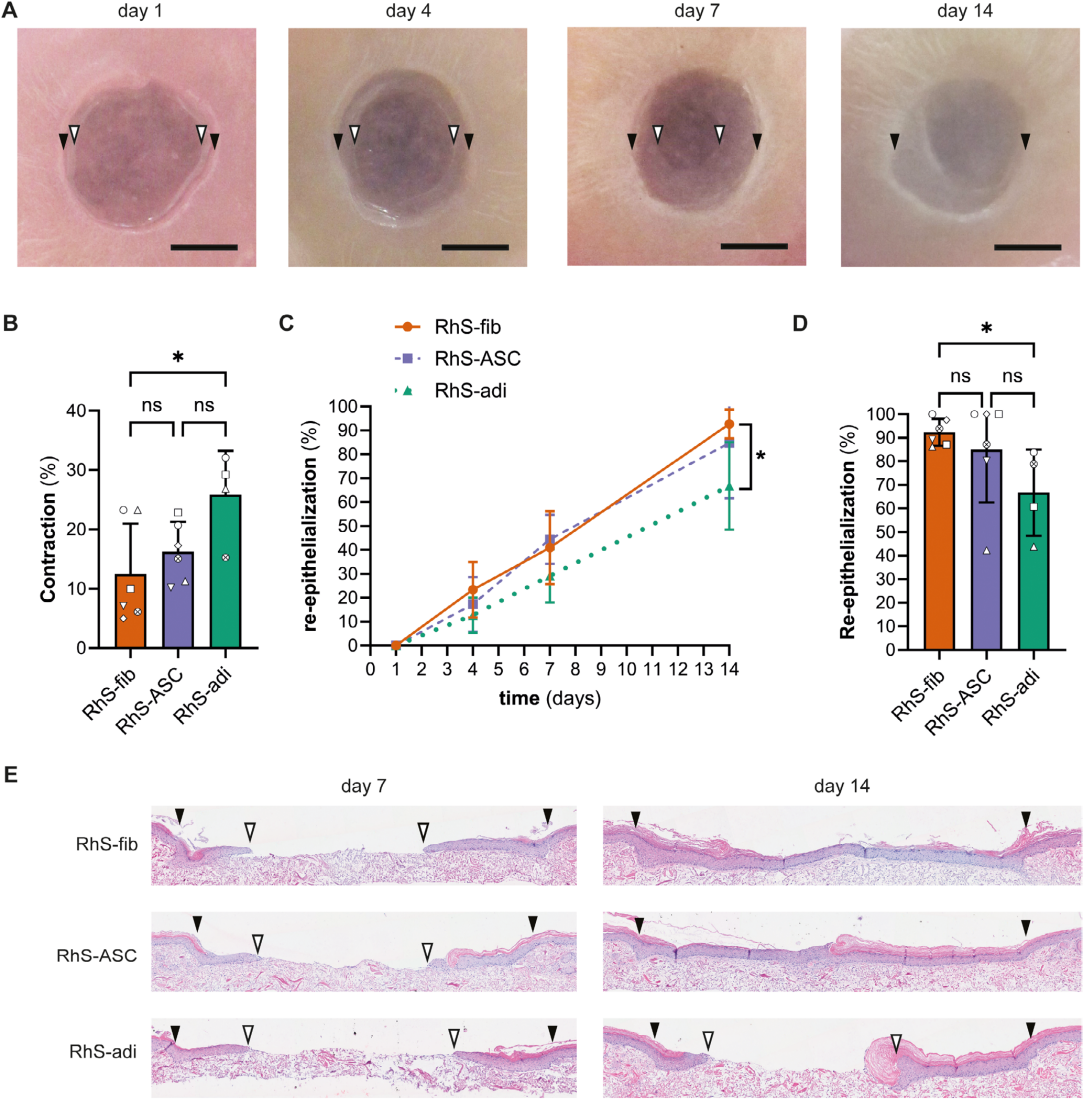
Re‐epithelialization and contraction in superficial and full thickness wounds. (A) Macroscopic image of wound at day 1, 4, 7 and 14 after wounding (scale bar: 2 mm). Black arrowheads indicate wound margin, white arrowheads indicate migrating front. (B) Quantification of wound contraction at day 14/wound surface area (based on wound margin, black arrowheads in panel A) at day 14/wound surface area (based on wound margin, black arrowheads in panel A) at day 0). (C) Quantification of re‐epithelialization over time (based on histology; (total epithelial ingrowth (length between black and white arrowheads in panel E)/total wound size (length between black arrowheads) × 100%). (D) Quantification of re‐epithelialization at day 14. (E) HE‐stained tissue section of wound illustrating re‐epithelialization at day 7 (left) and day 14 (right). Black arrowheads indicate wound margin, white arrowheads indicate migrating front. Each symbol represents 1 independent experiment (*n* = 4–6). Mixed‐effects analysis with Tukey's multiple comparison test (B, D) and ordinary 2‐way ANOVA with Tukey's multiple comparisons test (C) were used for statistical analysis. *p < 0.05; ns, not significant.

Three different wound models were constructed to study the role of stromal cells and adipocytes in deep and superficial wound healing. Each model had a standard RhS dermal and epidermal layer, in which 4 mm diameter wounds were made. These wounded RhS were then placed on different wound bed layers containing either fibroblasts (RhS‐fib), ASCs (RhS‐ASC) or a mixture of ASCs and ASC‐derived adipocytes (RhS‐adi) (Figure 1). Macroscopic evaluation of the wounds revealed gradual wound contraction and closure over a period of 14 days (Figure 3A). Interestingly, increased wound contraction was observed in the constructs with adipocytes in the wound bed but not when only ASCs were in the wound bed (Figure 3B). Histological evaluation of cross sections of the wounded area of all donors, including the separate technical replicates for each donor, revealed that in total, 8 out of 15 RhS‐fib constructs were completely re‐epithelialised 14 days after wounding. Similarly, 8 out of 15 RhS‐ASC constructs were completely re‐epithelialised, while only 1 out of 9 RhS‐adi constructs were completely healed after 14 days. When comparing the percentage of re‐epithelialisation for the different donors (with averaged technical replicate values) we observed a significant decrease in re‐epithelialisation in the RhS‐adi compared to RhS‐fib (Figure 3C–E). Overall, the lack of fibroblasts or the presence of adipocytes in the open wound bed and near the migrating front delayed re‐epithelialisation.

**FIGURE 4:**
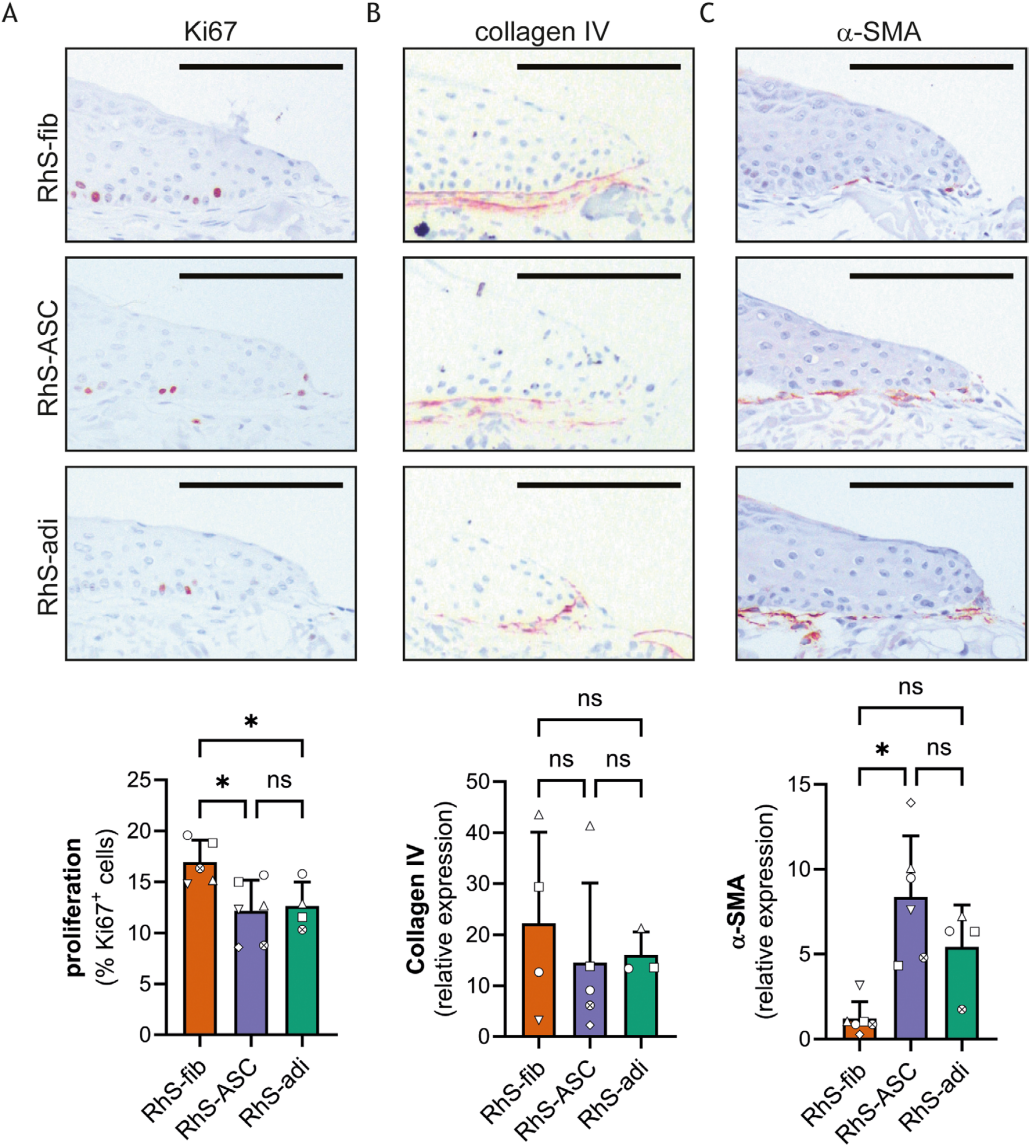
Re‐epithelialization in superficial and full thickness wounds details healing front. Representative pictures and quantification of IHC staining for Ki67 (A), collagen IV (B), and α‐SMA (C) at the migrating front in RhS‐fib, RhS‐ASC, and RhS‐adi. Scale bar is 100 μm. All migrating fronts (left and right side at each time point available) were used for analysis. Each symbol represents 1 independent experiment (*n* = 3–6). Mixed‐effects analysis with Geisser–Greenhouse correction and Tukey's multiple comparison tests was used for statistical analysis. *p < 0.05; ns, not significant.

Re‐epithelialisation is a complex and well‐coordinated process that involves migration and proliferation of keratinocytes and is regulated by cytokine crosstalk between epithelial and stromal cells [24, 25]. As shown in Figure 4A, the percentage of Ki67‐positive cells was reduced in the migrating epithelial front of RhS‐ASC and RhS‐adi, as compared to RhS‐fib. No significant difference in proliferation was found between RhS‐ASC and RhS‐adi cultures, suggesting that additional factors contribute to (reduced) re‐epithelialisation in RhS‐adi. The formation of a basement membrane is pivotal for keratinocyte migration during wound healing [26]. In all the wounds, a basement membrane was formed as indicated by collagen IV deposition at the dermal‐epidermal junction at the migrating front (Figure 4B). No significant difference in the amount of collagen IV was found between the different models. Next, α‐SMA expression, a marker for myofibroblast differentiation, was investigated. Interestingly, many α‐SMA‐positive fibroblasts were observed directly under the newly formed epidermis in RhS‐ASC, while no α‐SMA staining was observed in the regions next to the wound. The α‐SMA expression in the wound bed (underneath the migrating epithelium) was significantly increased in RhS‐ASC as compared to RhS‐fib (Figure 4C). A trend towards increased α‐SMA in RhS‐adi as compared to RhS‐fib and decreased α‐SMA in RhS‐adi as compared to RhS‐ASC was observed. These data indicate that the absence of fibroblasts in the wound bed of deep wound RhS results in diminished proliferation of keratinocytes. The presence of ASCs does improve re‐epithelialisation; however, this is accompanied by increased α‐SMA expression, suggesting increased scar formation.

### 3.3 Cytokine Expression in Deep and Superficial Wound Models

**FIGURE 5:**
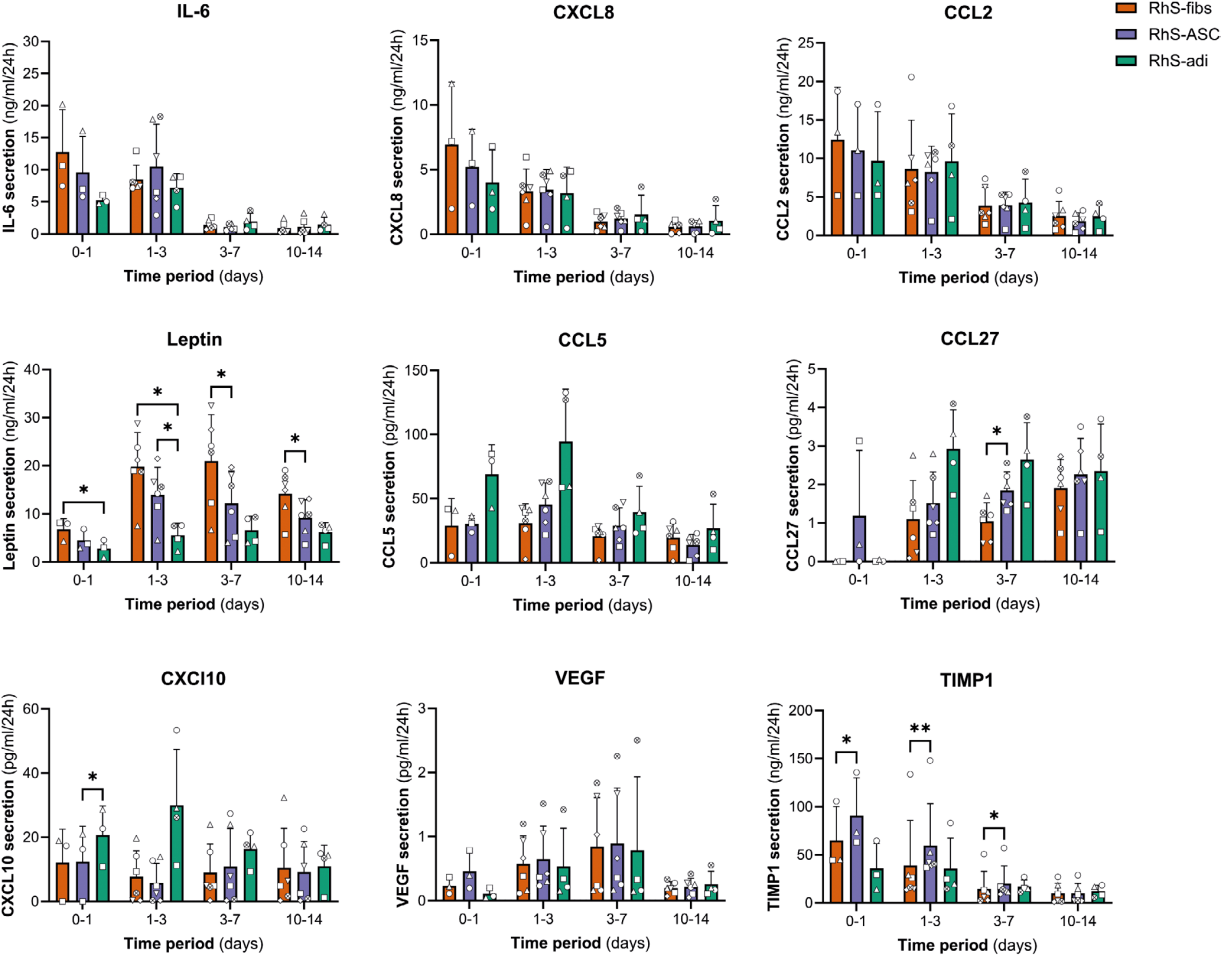
Cytokine secretion in RhS‐fib, RhS‐ASC, and RhS‐adi over time. Concentration of wound healing factors secreted by RhS‐fib, RhS‐adi, and RhS‐adi per 24 h 0–1, 1–3, 3–7, and 10–14 days after construction. Each symbol represents 1 independent experiment (*n* = 3–6). Mixed‐effects analysis with Geisser–Greenhouse correction and Tukey's multiple comparison tests was used for statistical analysis. *p < 0.05; **p < 0.01.

Lastly, we investigated a panel of inflammatory chemokines, cytokines, and growth factors that are involved in wound healing. Fibroblasts provide cytokines and growth factors such as IL‐6, CXCL8, leptin, and CCL2 required for keratinocytes to proliferate, migrate, and to produce basement membrane proteins such as collagen IV and laminin [25]. CCL5, CXCL10, and CCL27 are chemokines that are secreted by keratinocytes upon wounding and are chemoattractants for dermal fibroblasts, ASCs, and bone marrow‐derived stem cells, as well as immune cells such as T lymphocytes [21, 27, 28, 29]. VEGF and TIMP‐1 are required for wound healing and are prototypical markers for scar formation [30, 31]. Cytokine secretion was comparable in unwounded and wounded RhS‐fib, RhS‐ASC, and RhS‐adi models and followed a similar trend over time (data not shown), indicating that the differences in cytokine secretion observed are due to a combination of the cell types included in the model and the response of these cell types to the construction procedure, as well as the inflicted wound (Figure 1). However, it was noted that after construction, which included inflicting the wounds, leptin levels were increased in RhS‐fib as compared to RhS‐ASC and RhS‐adi at multiple time points and were also higher in RhS‐ASC compared to RhS‐adi between the time interval of 1–3 days. CCL27 was increased in RhS‐ASC as compared to RhS‐fib 3–7 days after construction. CXCL10 was increased in RhS‐adi as compared to RhS‐fib 1 day after construction, and TIMP‐1 was increased in RhS‐ASC compared to RhS‐fib up until 7 days after construction (Figure 5). No significant differences were found for IL‐6, CXCL8, CCL2, CCL5, and VEGF.

## 4 Discussion

In this study, we developed three different RhS models, representative of superficial wound healing and deep wound healing with and without adipocytes, that allow for the in vitro study of wound closure, contraction, α‐SMA expression and cytokine release over time. In accordance with clinical observations in superficial wounds, the superficial wound model RhS‐fib heals quickly and with little scar formation. The RhS‐adi model (consisting of both adipocytes and ASCs, representative of deep wound healing) shows reduced re‐epithelialisation capacity, increased contraction, and presence of myofibroblasts underneath the newly formed epithelium, indicative of scar formation. Although the RhS‐ASC model also healed with a considerable increase in myofibroblasts, re‐epithelialisation and contraction were similar to RhS‐fib. The presence of adipocytes in the model thus seems to promote the deep wound phenotype.

Our 3D wound healing models RhS‐fib, RhS‐ASC, and RhS‐adi showed similar keratinocyte proliferation rates at the unwounded areas, although this was slightly higher as compared to the proliferation rates of native epidermis and previously published RhS models (~18% vs. ~12%, respectively) [32, 33]. In our study, wounds are induced using a 4 mm diameter biopsy punch, resulting in a clean open acute wound. In our RhS‐fibs model, the proliferation rate at the wounded area was similar to that of unwounded areas, as well as to superficial RhS wounds made by us and others [34]. The wound healing process in our models resembles the healing of a clean surgery wound or debrided wound with limited cell damage. The rate of re‐epithelialisation is similar in the current clean surgery model as compared to previously described freeze wound healing models, but higher than the re‐epithelialisation rate of heat‐induced burn wound models [20, 24, 35]. As opposed to currently available wound models where damage is induced via heat or cold exposure, the clean and standardised punch wound does not develop a blister, and test substances can therefore be applied directly onto the wound bed in the future. Previously, we have shown that the blister resulting from freeze of burn wounding RhS in vitro limits the ability of topical actives to reach the wound bed and therefore is a major limitation in such models [20, 35].

Our RhS‐adi model is the first 3D RhS wound healing model that includes a subcutaneous fat layer. Bi‐ or tri‐layered models in which keratinocytes were seeded on top of ASCs or ASCs differentiated into adipocytes have been published before, but only as an intact skin model or end‐stage scar model [8, 14, 36, 37]. Similar to our results in this present study, van den Broek et al. and El‐Ghalbzouri et al. show α‐SMA expression at the dermal‐epidermal junction when keratinocytes are cultured on top of ASC‐populated matriderm or collagen hydrogel [8, 36]. α‐SMA expression was not observed in the model of Trottier et al. [37] where keratinocytes were cultured on top of self‐assembled sheets of ASCs. The difference in α‐SMA expression may be due to the different methodology used (self‐assembled dermal scaffold versus hydrogel or matriderm) or different culture media (e.g., added FCS or serum alternatives FetalClone II or UltroserG). Previously, we have shown that an RhS‐ASC model similar to the one described here, but without a wound, could be used to test drugs and actives which can be used to reduce existing scar characteristics [8]. Indeed, treatment with 5‐fluorouracil (also known as 5FU), a drug that is being used to treat scars in the clinic, resulted in reduced contraction in this scar‐RhS model [8]. Moreover, the model was used to identify atorvastatin as a potential novel scar therapeutic and statin use was later shown to be associated with reduced hypertrophic scar formation in a retrospective clinical study as well [8, 38]. In the current RhS‐ASC and RhS‐adi models, we can now investigate the effect of topical exposure of existing and novel drugs and actives on very early scar formation before the wound is closed. Such test substances could be considered as preventing scar formation rather than reducing or correcting existing scar properties. This is of importance since we have previously shown that the scar quality is already indicated directly upon wounding [39].

Re‐epithelialisation is a complex process that is highly dependent on the interaction between keratinocytes and the stromal cells underneath. In deep wounds where the dermis is completely gone, the lack of dermal fibroblasts is suggested to cause delayed re‐epithelialisation [24]. Indeed, transplantation of a dermal substitute with fibroblasts improved re‐epithelialisation in partial‐thickness burn wounds [40]. In the deep wound model RhS‐adi, a reduced re‐epithelialisation was observed as compared to RhS‐fib. In line, RhS‐adi showed reduced numbers of Ki67‐positive keratinocytes. Likely, autocrine signalling between the cells in the wound bed and keratinocytes plays an important role in wound closure and scar formation. Regarding cytokine secretion, only differences between the experimental conditions RhS‐fib, RhS‐ASC, and RhS‐adi were relevant. It was difficult to gain additional relevant information regarding cytokine release before and after wounding and over time due to the technical steps involved in constructing the models, most probably overriding the effect in cytokine secretion. This was because: (1) the punch biopsy wound removed an area of 7 mm^2^ of living cells which actively secreted cytokines (~5% of RhS surface); (2) the acute excision wound created by the punch only had a circumference of 19 mm which in comparison to the entire culture area of ~144 mm^2^ only represented a very small number of living cells which were damaged; (3) the construction of the models which involved transferring the dermal part onto the adipose or deep dermal part involved removing it from the transwell which in itself would result in trauma and cytokine release. New techniques, such as in situ sampling of localised cytokine release, may provide more insight into the autocrine signalling via cytokines and chemokines in the wound bed and near the migrating front in the future [41]. However, when sampling the supernatant underneath the RhS cultures, we did find significant differences in leptin, CCL27, CXCL10, and TIMP1 levels. Especially the finding that leptin levels were lowest in RhS‐adi was surprising, since leptin is one of the best‐described adipokines and is produced in adipose tissue. Both ASCs and fibroblasts produce leptin [42, 43], but no comparative studies have been performed as far as we know. All constructs were cultured in the same ALI medium (with 100 nM insulin, which stimulates leptin secretion) during the entire wound healing period. It is possible that the fibroblasts responded to insulin more than ASC or adipocytes, or that ASC or adipocytes take up more leptin as compared to fibroblasts.

An important limitation of the presented models is the lack of immune cells and the vascular system. Endothelial cells, neutrophils, macrophages and other immune cells play an important role in wound healing and scar formation. Cytokines measured in our model are all produced by non‐haematopoietic cells and represent the initiation of the immune response directly after wounding and do not reflect the cytokine milieu in a healing or homeostatic tissue in vivo. Influx of immune cells from the circulation, such as monocytes that differentiate into macrophages, for example, is an important source of IL‐6 during later stages of wound healing. IL‐6 stimulates TGF‐β secretion and myofibroblast differentiation and survival in the proliferation and remodelling phases of wound healing [44, 45]. The RhS‐ASC and RhS‐adi models indicate that myofibroblast differentiation and consequent scar formation do occur in the absence of immune cells such as macrophages. However, it is expected that the scar phenotypes will become more exaggerated with the incorporation of immune cells, in particular macrophages, which are predominant cells in adult skin wounds [46]. T cells, macrophages and neutrophils are currently being incorporated in different 3D skin models and may be added to these 3D wound healing models in the future as well [47].

In conclusion, our models offer a unique, human‐based testing platform to further investigate the interaction between adipocytes, different stromal cell types, and epithelium during wound re‐epithelialisation and contraction and can be used to test the topical application of drugs applied directly into an open wound. They can be used to simultaneously study re‐epithelialisation, contraction, and cytokine secretion in a standardised setting.

## Author Contributions

Conceptualisation: S.G., M.W., and I.V. Funding acquisition: S.G. Investigation: M.W., J.P.N.‐B., and S.R. Methodology: I.V. and M.W. Visualisation: M.W. Writing – original draft preparation: M.W. and S.G. Writing – review and editing: M.W., S.G., and J.P.N.‐B.

## Ethics Statement

Adult human skin with adipose tissue was obtained from healthy donors as rest material after abdominal dermolipectomy. Tissue was collected after informed consent and in anonymous fashion in accordance with the “Human Tissue and Medical Research: Code of conduct for responsible use” formulated by the Foundation Federation of Dutch Medical Scientific Societies.

## Conflicts of Interest

The authors declare no conflicts of interest.

## Supporting information


**Figure S1.** wrr70047‐sup‐0001‐Supinfo.

## Data Availability

The data that support the findings of this study are available from the corresponding author upon reasonable request.

## References

[1] C. S. J. Dunkin , J. M. Pleat , P. H. Gillespie , M. P. H. Tyler , A. H. N. Roberts , and D. A. McGrouther , “Scarring Occurs at a Critical Depth of Skin Injury: Precise Measurement in a Graduated Dermal Scratch in Human Volunteers,” Plastic and Reconstructive Surgery 119, no. 6 (2007): 1722–1732, 10.1097/01.prs.0000258829.07399.f0. 17440346

[2] C. C. Finnerty , M. G. Jeschke , L. K. Branski , J. P. Barret , P. Dziewulski , and D. N. Herndon , “Hypertrophic Scarring: The Greatest Unmet Challenge After Burn Injury,” Lancet 388, no. 10052 (2016): 1427–1436, 10.1016/S0140-6736(16)31406-4. 27707499 PMC5380137

[3] M. Waasdorp , B. P. Krom , F. J. Bikker , P. P. M. van Zuijlen , F. B. Niessen , and S. Gibbs , “The Bigger Picture: Why Oral Mucosa Heals Better Than Skin,” Biomolecules 11, no. 8 (2021): 1165, 10.3390/biom11081165. 34439831 PMC8394648

[4] L. M. G. Neves , T. A. Wilgus , and A. Bayat , “In Vitro, Ex Vivo, and In Vivo Approaches for Investigation of Skin Scarring: Human and Animal Models,” Advances in Wound Care 12, no. 2 (2023): 97–116, 10.1089/wound.2021.0139. 34915768

[5] H. H. Sami DGH and A. Abdellatif , “Wound Healing Models: A Systematic Review of Animal and Non‐Animal Models,” Wound Medicine 24, no. 1 (2019): 10.

[6] H. D. Zomer and A. G. Trentin , “Skin Wound Healing in Humans and Mice: Challenges in Translational Research,” Journal of Dermatological Science 90, no. 1 (2018): 3–12, 10.1016/j.jdermsci.2017.12.009. 29289417

[7] G. C. Limandjaja , L. J. van den Broek , M. Breetveld , et al., “Characterization of In Vitro Reconstructed Human Normotrophic, Hypertrophic, and Keloid Scar Models,” Tissue Engineering. Part C, Methods 24, no. 4 (2018): 242–253, 10.1089/ten.TEC.2017.0464. 29490604

[8] L. J. van den Broek , F. B. Niessen , R. J. Scheper , and S. Gibbs , “Development, Validation and Testing of a Human Tissue Engineered Hypertrophic Scar Model,” ALTEX 29, no. 4 (2012): 389–402, 10.14573/altex.2012.4.389. 23138509

[9] M. L. Zou , Y. Y. Teng , J. J. Wu , et al., “Fibroblasts: Heterogeneous Cells With Potential in Regenerative Therapy for Scarless Wound Healing,” Frontiers in Cell and Development Biology 9 (2021): 713605, 10.3389/fcell.2021.713605. PMC8329665 34354997

[10] R. R. Driskell , B. M. Lichtenberger , E. Hoste , et al., “Distinct Fibroblast Lineages Determine Dermal Architecture in Skin Development and Repair,” Nature 504, no. 7479 (2013): 277–281, 10.1038/nature12783. 24336287 PMC3868929

[11] D. Correa‐Gallegos , D. Jiang , S. Christ , et al., “Patch Repair of Deep Wounds by Mobilized Fascia,” Nature 576, no. 7786 (2019): 287–292, 10.1038/s41586-019-1794-y. 31776510

[12] D. Jiang , S. Christ , D. Correa‐Gallegos , et al., “Injury Triggers Fascia Fibroblast Collective Cell Migration to Drive Scar Formation Through N‐Cadherin,” Nature Communications 11, no. 1 (2020): 5653, 10.1038/s41467-020-19425-1. PMC7648088 33159076

[13] B. A. Shook , R. R. Wasko , O. Mano , et al., “Dermal Adipocyte Lipolysis and Myofibroblast Conversion Are Required for Efficient Skin Repair,” Cell Stem Cell 26, no. 6 (2020): 880–895, 10.1016/j.stem.2020.03.013. 32302523 PMC7853423

[14] J. Jager , I. Vahav , M. Thon , et al., “Reconstructed Human Skin With Hypodermis Shows Essential Role of Adipose Tissue in Skin Metabolism,” Tissue Engineering and Regenerative Medicine 21 (2024): 499–511, 10.1007/s13770-023-00621-1. 38367122 PMC10987437

[15] M. Blais , L. Mottier , M. A. Germain , S. Bellenfant , S. Cadau , and F. Berthod , “Sensory Neurons Accelerate Skin Reepithelialization via Substance P in an Innervated Tissue‐Engineered Wound Healing Model,” Tissue Engineering. Part A 20, no. 15–16 (2014): 2180–2188, 10.1089/ten.tea.2013.0535. 24716723 PMC4137331

[16] C. M. Catarino , T. do Nascimento Pedrosa , P. C. Pennacchi , et al., “Skin Corrosion Test: A Comparison Between Reconstructed Human Epidermis and Full Thickness Skin Models,” European Journal of Pharmaceutics and Biopharmaceutics 125 (2018): 51–57, 10.1016/j.ejpb.2018.01.002. 29317274

[17] S. Kuchler , N. B. Wolf , S. Heilmann , et al., “3D‐Wound Healing Model: Influence of Morphine and Solid Lipid Nanoparticles,” Journal of Biotechnology 148, no. 1 (2010): 24–30, 10.1016/j.jbiotec.2010.01.001. 20138929

[18] A. F. Laplante , L. Germain , F. A. Auger , and V. Moulin , “Mechanisms of Wound Reepithelialization: Hints From a Tissue‐Engineered Reconstructed Skin to Long‐Standing Questions,” FASEB Journal 15, no. 13 (2001): 2377–2389, 10.1096/fj.01-0250com. 11689463

[19] V. J. Moulin , J. Dube , O. Rochette‐Drouin , et al., “Electric Potential Across Epidermis and Its Role During Wound Healing Can be Studied by Using an In Vitro Reconstructed Human Skin,” Advances in Wound Care 1, no. 2 (2012): 81–87, 10.1089/wound.2011.0318. 24527285 PMC3839018

[20] C. Rodrigues Neves , J. Buskermolen , S. Roffel , et al., “Human Saliva Stimulates Skin and Oral Wound Healing In Vitro,” Journal of Tissue Engineering and Regenerative Medicine 13, no. 6 (2019): 1079–1092, 10.1002/term.2865. 30968584 PMC6593997

[21] K. L. Kroeze , W. J. Jurgens , B. Z. Doulabi , F. J. van Milligen , R. J. Scheper , and S. Gibbs , “Chemokine‐Mediated Migration of Skin‐Derived Stem Cells: Predominant Role for CCL5/RANTES,” Journal of Investigative Dermatology 129, no. 6 (2009): 1569–1581, 10.1038/jid.2008.405. 19122644

[22] T. Waaijman , M. Breetveld , M. Ulrich , E. Middelkoop , R. J. Scheper , and S. Gibbs , “Use of a Collagen‐Elastin Matrix as Transport Carrier System to Transfer Proliferating Epidermal Cells to Human Dermis In Vitro,” Cell Transplantation 19, no. 10 (2010): 1339–1348, 10.3727/096368910X507196. 20525428

[23] M. J. Lee and S. K. Fried , “Optimal Protocol for the Differentiation and Metabolic Analysis of Human Adipose Stromal Cells,” Methods in Enzymology 538 (2014): 49–65, 10.1016/B978-0-12-800280-3.00004-9. 24529433 PMC4336794

[24] A. El Ghalbzouri , P. Hensbergen , S. Gibbs , J. Kempenaar , R. van der Schors , and M. Ponec , “Fibroblasts Facilitate Re‐Epithelialization in Wounded Human Skin Equivalents,” Laboratory Investigation 84, no. 1 (2004): 102–112, 10.1038/labinvest.3700014. 14631386

[25] B. Russo , N. C. Brembilla , and C. Chizzolini , “Interplay Between Keratinocytes and Fibroblasts: A Systematic Review Providing a New Angle for Understanding Skin Fibrotic Disorders,” Frontiers in Immunology 11 (2020): 648, 10.3389/fimmu.2020.00648. 32477322 PMC7232541

[26] P. Rousselle , M. Montmasson , and C. Garnier , “Extracellular Matrix Contribution to Skin Wound Re‐Epithelialization,” Matrix Biology 75‐76 (2019): 12–26, 10.1016/j.matbio.2018.01.002. 29330022

[27] P. A. Rees , N. S. Greaves , M. Baguneid , and A. Bayat , “Chemokines in Wound Healing and as Potential Therapeutic Targets for Reducing Cutaneous Scarring,” Advances in Wound Care 4, no. 11 (2015): 687–703, 10.1089/wound.2014.0568. 26543682 PMC4620529

[28] A. Ridiandries , J. T. M. Tan , and C. A. Bursill , “The Role of Chemokines in Wound Healing,” International Journal of Molecular Sciences 19, no. 10 (2018): 3217, 10.3390/ijms19103217. 30340330 PMC6214117

[29] S. W. Spiekstra , M. Breetveld , T. Rustemeyer , R. J. Scheper , and S. Gibbs , “Wound‐Healing Factors Secreted by Epidermal Keratinocytes and Dermal Fibroblasts in Skin Substitutes,” Wound Repair and Regeneration 15, no. 5 (2007): 708–717, 10.1111/j.1524-475X.2007.00280.x. 17971017

[30] D. Ulrich , F. Ulrich , F. Unglaub , A. Piatkowski , and N. Pallua , “Matrix Metalloproteinases and Tissue Inhibitors of Metalloproteinases in Patients With Different Types of Scars and Keloids,” Journal of Plastic, Reconstructive and Aesthetic Surgery 63, no. 6 (2010): 1015–1021, 10.1016/j.bjps.2009.04.021. 19464975

[31] T. A. Wilgus , “Vascular Endothelial Growth Factor and Cutaneous Scarring,” Advances in Wound Care 8, no. 12 (2019): 671–678, 10.1089/wound.2018.0796. 31750015 PMC6862968

[32] M. A. Boink , S. Roffel , M. Breetveld , et al., “Comparison of Advanced Therapy Medicinal Product Gingiva and Skin Substitutes and Their In Vitro Wound Healing Potentials,” Journal of Tissue Engineering and Regenerative Medicine 12, no. 2 (2018): e1088–e1097, 10.1002/term.2438. 28388010 PMC5836907

[33] S. Gibbs , J. Vicanova , J. Bouwstra , D. Valstar , J. Kempenaar , and M. Ponec , “Culture of Reconstructed Epidermis in a Defined Medium at 33 Degrees C Shows a Delayed Epidermal Maturation, Prolonged Lifespan and Improved Stratum Corneum,” Archives of Dermatological Research 289, no. 10 (1997): 585–595, 10.1007/s004030050244. 9373718

[34] L. I. S. Naasani , J. Sevigny , V. J. Moulin , and M. R. Wink , “UTP Increases Wound Healing in the Self Assembled Skin Substitute (SASS),” Journal of Cell Communication and Signaling 17, no. 3 (2023): 827–844, 10.1007/s12079-023-00725-2. 36723784 PMC10409941

[35] M. Breetveld , C. D. Richters , T. Rustemeyer , R. J. Scheper , and S. Gibbs , “Comparison of Wound Closure After Burn and Cold Injury in Human Skin Equivalents,” Journal of Investigative Dermatology 126, no. 8 (2006): 1918–1921, 10.1038/sj.jid.5700330. 16645585

[36] A. El‐Ghalbzouri , A. J. Van Den Bogaerdt , J. Kempenaar , and M. Ponec , “Human Adipose Tissue‐Derived Cells Delay Re‐Epithelialization in Comparison With Skin Fibroblasts in Organotypic Skin Culture,” British Journal of Dermatology 150, no. 3 (2004): 444–454, 10.1046/j.1365-2133.2004.05830.x. 15030326

[37] V. Trottier , G. Marceau‐Fortier , L. Germain , C. Vincent , and J. Fradette , “IFATS Collection: Using Human Adipose‐Derived Stem/Stromal Cells for the Production of New Skin Substitutes,” Stem Cells 26, no. 10 (2008): 2713–2723, 10.1634/stemcells.2008-0031. 18617689

[38] C. Chello , A. Nenna , M. Chello , et al., “Statin Treatment and Hypertrophic Scarring After Cardiac Surgery,” Wound Repair and Regeneration 29, no. 1 (2021): 129–133, 10.1111/wrr.12878. 33236817

[39] L. Butzelaar , D. P. Schooneman , E. A. Soykan , et al., “Inhibited Early Immunologic Response Is Associated With Hypertrophic Scarring,” Experimental Dermatology 25, no. 10 (2016): 797–804, 10.1111/exd.13100. 27249786

[40] R. J. Kumar , R. M. Kimble , R. Boots , and S. P. Pegg , “Treatment of Partial‐Thickness Burns: A Prospective, Randomized Trial Using Transcyte,” ANZ Journal of Surgery 74, no. 8 (2004): 622–626, 10.1111/j.1445-1433.2004.03106.x. 15315558

[41] E. Michielon , A. C. Motta , J. Ogien , et al., “Integration of Line‐Field Confocal Optical Coherence Tomography and In Situ Microenvironmental Mapping to Investigate the Living Microenvironment of Reconstructed Human Skin and Melanoma Models,” Journal of Dermatological Science 115, no. 2 (2024): 85–93, 10.1016/j.jdermsci.2024.07.001. 39043504

[42] A. Glasow , W. Kiess , U. Anderegg , A. Berthold , A. Bottner , and J. Kratzsch , “Expression of Leptin (Ob) and Leptin Receptor (Ob‐R) in Human Fibroblasts: Regulation of Leptin Secretion by Insulin,” Journal of Clinical Endocrinology and Metabolism 86, no. 9 (2001): 4472–4479, 10.1210/jcem.86.9.7792. 11549696

[43] A. L. Strong , T. A. Strong , L. V. Rhodes , et al., “Obesity Associated Alterations in the Biology of Adipose Stem Cells Mediate Enhanced Tumorigenesis by Estrogen Dependent Pathways,” Breast Cancer Research 15, no. 5 (2013): R102, 10.1186/bcr3569. 24176089 PMC3978929

[44] G. Casella , L. Garzetti , A. T. Gatta , et al., “IL4 Induces IL6‐Producing M2 Macrophages Associated to Inhibition of Neuroinflammation In Vitro and In Vivo,” Journal of Neuroinflammation 13, no. 1 (2016): 139, 10.1186/s12974-016-0596-5. 27266518 PMC4895901

[45] B. Z. Johnson , A. W. Stevenson , C. M. Prele , M. W. Fear , and F. M. Wood , “The Role of IL‐6 in Skin Fibrosis and Cutaneous Wound Healing,” Biomedicine 8, no. 5 (2020): 101, 10.3390/biomedicines8050101. PMC7277690 32365896

[46] L. A. DiPietro , T. A. Wilgus , and T. J. Koh , “Macrophages in Healing Wounds: Paradoxes and Paradigms,” International Journal of Molecular Sciences 22, no. 2 (2021): 950, 10.3390/ijms22020950. 33477945 PMC7833402

[47] P. P. G. Mulder , M. Vlig , A. Elgersma , et al., “Monocytes and T Cells Incorporated in Full Skin Equivalents to Study Innate or Adaptive Immune Reactions After Burn Injury,” Frontiers in Immunology 14 (2023): 1264716, 10.3389/fimmu.2023.1264716. 37901218 PMC10611519

