# Supplementary material for: Reconstructed Human Skin Models to Study Superficial and Deep Skin Wound Healing In Vitro
Source: Wound Repair Regen. 2025 Jun 5;33(3):e70047. doi: 10.1111/wrr.70047 (PMC12138859; doi:10.1111/wrr.70047)
Supplement: Supplementary file 1 — Figure S1. wrr70047‐sup‐0001‐Supinfo. [file WRR-33-0-s001.docx]

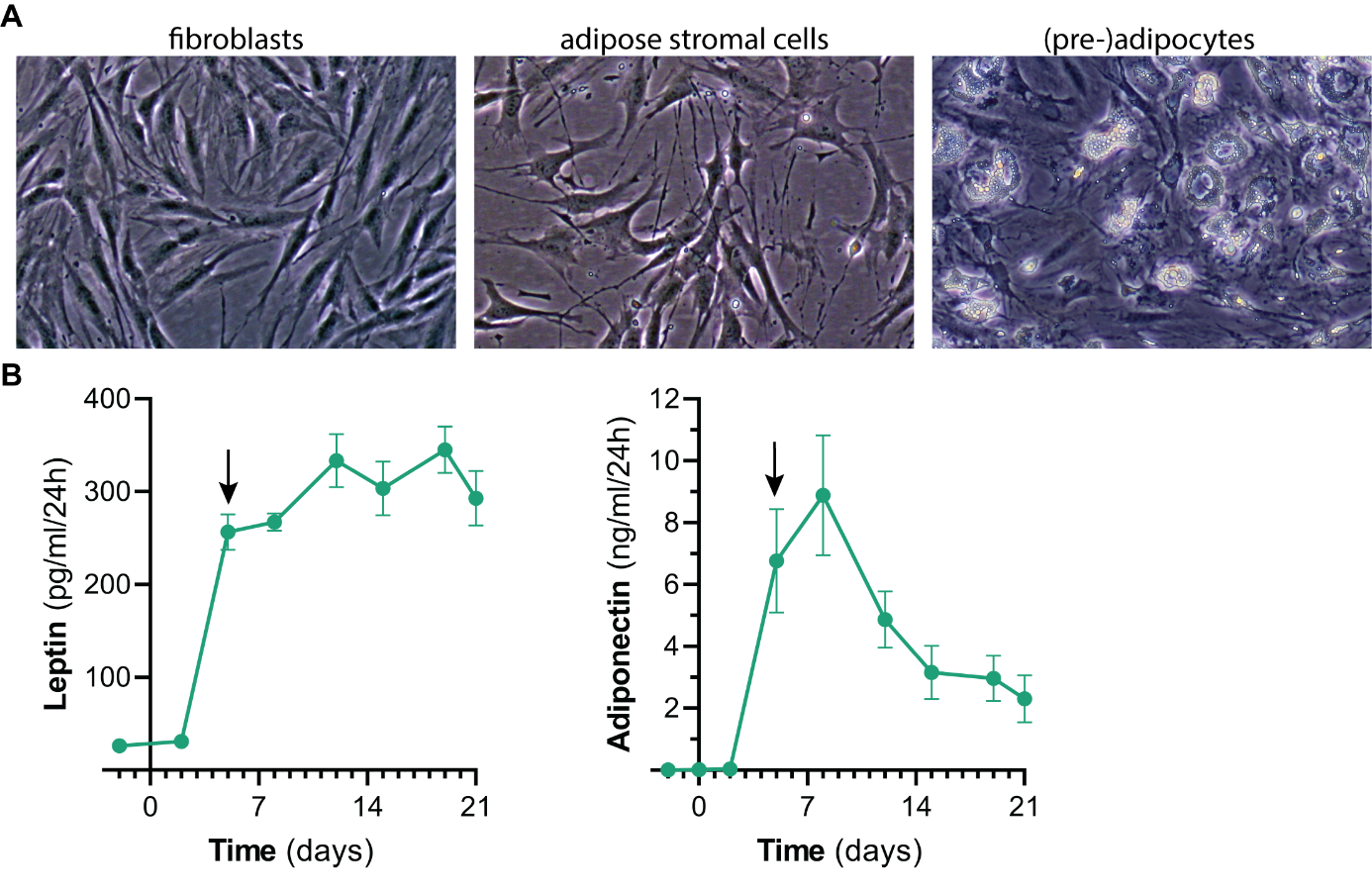


**Supplementary figure 1: Differentiation of adipose stromal cells into (pre-)adipocytes.** (A) Representative pictures at 200x magnification illustrating fibroblasts, ASCs and (pre)adipocyte morphology prior to seeding in Matriderm. (B) Leptin and adiponectin secretion in culture supernatant during adipocyte differentiation. Arrows indicate the timepoint at which pre-adipocytes were harvested and seeded onto Matriderm for further maturation inside the 3D collagen/elastin matrix.
